# Fibrin Sealants in Dura Sealing: A Systematic Literature Review

**DOI:** 10.1371/journal.pone.0151533

**Published:** 2016-04-27

**Authors:** Felice Esposito, Filippo Flavio Angileri, Peter Kruse, Luigi Maria Cavallo, Domenico Solari, Vincenzo Esposito, Francesco Tomasello, Paolo Cappabianca

**Affiliations:** 1 Department of Biomedical and Dental Sciences and Morpho-Functional Imaging, Division of Neurosurgery, Università degli Studi di Messina, Messina, Italy; 2 Independent medical advisor, Cervignano del Friuli, Italy; 3 Department of Neurosciences and Odontostomatological and Reproductive Sciences, Division of Neurosurgery, Università degli Studi di Napoli Federico II, Naples, Italy; 4 Division of Neurosurgery, Università degli Studi di Roma Sapienza, Rome, Italy; Universita degli Studi di Palermo, ITALY

## Abstract

**Background:**

Fibrin sealants are widely used in neurosurgery to seal the suture line, provide watertight closure, and prevent cerebrospinal fluid leaks. The aim of this systematic review is to summarize the current efficacy and safety literature of fibrin sealants in dura sealing and the prevention/treatment of cerebrospinal fluid leaks.

**Methods:**

A comprehensive electronic literature search was run in the following databases: Cochrane Database of Systematic Reviews, Cochrane Central Resister of Controlled Trials, clinicaltrials.gov, MEDLINE/PubMed, and EMBASE. Titles and abstracts of potential articles of interest were reviewed independently by 3 of the authors.

**Results:**

A total of 1006 database records and additional records were identified. After screening for duplicates and relevance, a total of 78 articles were assessed by the investigators for eligibility. Thirty-eight were excluded and the full-text of 40 articles were included in the qualitative synthesis. Seven of these included only safety data and were included in the safety assessment. The remaining 33 articles included findings from 32 studies that enrolled a total of 2935 patients who were exposed to fibrin sealant. Among these 33 studies there were only 3 randomized controlled trials, with the remaining being prospective cohort analysis, case controlled studies, prospective or retrospective case series. One randomized controlled trial, with 89 patients exposed to fibrin sealant, found a greater rate of intraoperative watertight dura closure in the fibrin sealant group than the control group (92.1% versus 38.0%, p<0.001); however, post-operative cerebrospinal fluid leakage occurred in more fibrin sealant than control patients (6.7% versus 2.0%, p>0.05). Other clinical trials evaluated the effect of fibrin sealant in the postoperative prevention of cerebrospinal fluid leaks. These were generally lower level evidence studies (ie, not prospective, randomized, controlled trials) that were not designed or powered to demonstrate a significant advantage to fibrin sealant use. Two small case series studies evaluated the effect of fibrin sealants in persistent cerebrospinal fluid leak treatment, but did not establish firm efficacy conclusions. Specific adverse reports where fibrin sealants were used for dura sealing were limited, with only 8 cases reported in neurosurgical procedures since 1987 and most reporting only a speculative relationship/association with fibrin sealant exposure.

**Conclusions:**

A major finding of this systematic literature review is that there is a paucity of randomized studies that have evaluated the effectiveness and safety of fibrin sealants in providing intraoperative watertight dura closure and post-operative cerebrospinal fluid leakage. Among the limited studies available, evidence from a single randomized, controlled trial indicates that fibrin sealants provide a higher rate of intraoperative watertight closure of the dura suture line than control, albeit with a higher rate of postoperative cerebrospinal fluid leakage. Evidence from non-randomized, controlled trials suggests that fibrin sealants may be effective in preventing cerebrospinal fluid leaks with an acceptable safety profile. There is a substantial need for randomized, controlled clinical trials or well-designed prospective observational trials where the conduct of a randomized trial is not feasible to fully assess the impact of fibrin sealant utilization on the rates of intraoperative dura closure, postoperative cerebrospinal leakage, and safety.

## Introduction

In neurosurgery, disruption of the arachnoid and dura allows cerebrospinal fluid (CSF) to flow or leak into an extradural space—an event that may lead to life-threatening complications such as infection and pneumocephalus. Reported incidences of CSF leaks vary from 0.8% to 13% depending on type of surgery and other factors [1–4]. The relative risk of developing meningitis in patients with postoperative CSF leak has been reported to be more than 10 [5].

Neurosurgeons aim to reduce the risk of CSF leaks by meticulous suturing of the dura, fibrin sealants, and by the use of dural patches/grafts when possible. In other cases, such as endonasal endoscopic skull base surgery, primary suturing of the dura mater may be very difficult or even impossible to achieve [6–11]. With increased intracranial pressure (>20 cm H2O), CSF may leak through even small suture holes causing a persistent CSF leak. To minimize the risk of such CSF leaks, new approaches have been applied to provide a watertight closure of the dural incision/lesion; one such treatment modality is to use a fibrin sealant (FS) directly on the suture line to seal the suture holes.

Fibrin sealants are commercially available products that contain two main active ingredients, fibrinogen and thrombin (human/animal/recombinant origin), that when mixed form a fibrin clot [12]. These products are used to seal biological tissues, either as two component (ie, fibrinogen and thrombin) liquid glue or as a two component dry patch. Liquid glue formulations include Tisseel^®^ or Tissucol^™^ (Baxter, Deerfield, IL, USA), Evicel^®^ (Ethicon US, LLC), and dry patch products such as Tachosil^®^ (Baxter, Deerfield, IL, USA) and Tachocomb^®^ (CSL Behring, Tokyo, Japan) [12]. Among these products, Tisseel / Tissucol is one of the first fibrin sealants and has been on the market over 30 years—it was first introduced to the European market in 1978. Tisseel has two main active component groups: 1) Human fibrinogen, synthetic aprotinin (anti fibrinolytic) and Factor XIII and 2) Human thrombin and calcium chloride [9]. When the two groups are mixed and applied to tissue, the fibrinogen and thrombin lead to generation of a fibrin clot that will adhere to tissue and have haemostatic (cease bleeding) and adhesive (seal and act as a bio glue) capacities [13].

The haemostatic effectiveness of using a fibrin sealant in surgery has been well established in a Cochrane review establishing its reduction of both postoperative blood loss and perioperative exposure to allogeneic red blood cell transfusion [14]. The non-blood compartment air tight and water tight sealing effects of fibrin sealants have been studied in randomized controlled trials of many types of surgery. These include sealing of intestinal anastomosis and preventing anastomotic leaks [12, 15–17], reduction of alveolar air leaks after pulmonary surgery [18], prevention of urethrocutaneous fistula after hypospadias repair [19], and in prevention of pancreatic fistula [20] although an earlier randomized trial did not report this same effect [21]. Fistulae sealing represents a therapeutic use of fibrin sealants with studies reporting fistulae closure in perianal/anal fistulaes [22, 23], complex anal fistulae [24, 25], and obstetrical vesico-vaginal fistulaes [26].

In patients undergoing neurosurgical procedures, fibrin sealant properties of tissue adhesion, sealing, and bioabsorption potentially provide logical and clinically relevant benefits in where there is a need for effective dura mater sealing, prevention of perioperative acute CSF leakage, and/or treatment of persistent postoperative CSF leakage. The aim of this systematic review is to evaluate and summarize the current dura sealing efficacy and safety literature of fibrin sealants. This information will provide guidance as to whether the clinical efficacy and safety data for fibrin sealants support their use in dura sealing, either to prevent acute CSF perioperative leaks in patients following neurosurgery or to prevent/treat persistent CSF postoperative/post-instrumentation leaks in patients undergoing neurologic or spinal procedures.

## Methods

### Search Strategy

PRISMA recommendations and criteria for a structured literature search and review were followed [27]. An extensive systematic literature search was performed by an independent clinician (PK) of the following electronic databases: Cochrane Database of Systematic Reviews (http://www.cochrane.org/cochrane-reviews), Cochrane Central Register of Controlled Trials, MEDLINE/PubMed (1950 to present [2014 May]), www.clinicaltrials.gov (searched for on-going trials or other concluded trials), and EMBASE via Ovid (1974 to present [2014 May]).

For the MEDLINE and EMBASE searches, a wide variety of Medical Subject Headings (MeSH) have been used in key papers, therefore, exploded MeSH terms were used to ensure that all the appropriate papers were captured. The main search terms used to capture all the studies available in MEDLINE and EMBASE are summarized in Table 1. The Cochrane Library was searched using the search terms “fibrin sealant” or “cerebrospinal fluid.” For the www.clinicaltrials.gov search, the broad search terms of “dura”, “fibrin sealant”, or “CSF leak” were used to identify completed or ongoing clinical trials evaluating the use of fibrin sealants in dura sealing.

**Table 1:** Search Strategy for the MEDLINE/PubMed Database.

Number	Searches
Identification of procedures and treatment target (CSF leak):	
1	Neurosurgical Procedures [MeSH]
2	Neurosurgery [MeSH]
3	Cerebrospinal Fluid [MeSH]
4	Cerebrospinal fluid leak [MeSH]
5	cerebrospinal
6	cerebrospinal fluid
7	cerebrospinal fluid leak
8	dura mater
9	spinal puncture
10	spinal tap
11	#1 OR #2 OR #3 OR #4 OR #5 OR #6 OR #7 OR #8 OR #9 OR #10
Identification of relevant fibrin sealants:	
12	Fibrin Tissue Adhesive [MeSH]
13	fibrin adhesive
14	fibrin glue
15	fibrin seal
16	biological glue
17	biological seal
18	beriplast
19	biocol
20	collaseal
21	crosseal
22	evicel
23	hemaseal
24	omrixil
25	quixil
26	tachocomb
27	tachosil
28	tisseel
29	tissel
30	tissucol
31	transglutine
32	vivostat
33	#12 OR #13 OR……..#32
Filter identification	
34	animals [mh] NOT humans [mh] Final search
35	#11 AND #33 NOT #34

### Article Eligibility and Selection

Two physicians (FE and PK) scanned independently the retrieved articles’ titles and abstracts for potential relevance and review inclusion eligibility. To be included, the article had to meet strict criteria, as listed in Table 2, with the search and inclusion criteria primarily targeting published studies or case reports presenting clinical efficacy and/or safety types of evaluations of fibrin sealants used intraoperatively during neurosurgical procedures to prevent and/or treat CSF leaks. If only a few or no randomized controlled trials were identified, lower evidence level reports (as defined in Table 3 [28]) were allowed for an evaluation of efficacy provided that the scope of the research was to evaluate the effect of fibrin sealants in dura sealing. The results of the two independent searches were matched in order to find the common results; the two physicians reviewed the unmatched findings once more, in order to check if they met the inclusion eligibility criteria. No cases of further disagreement between the two searching physicians occurred. Should any disagreement occurred, the relative articles would have been omitted from the analysis.

**Table 2:** Article Inclusion and Exclusion Criteria.

	Inclusion Criteria	Exclusion Criteria
Types of Studies	Randomized controlled trials evaluating the efficacy of fibrin sealants in dura sealing. Non-randomized, controlled trials reporting efficacy were allowed provided that the scope of the research was to evaluate the effect of fibrin sealants in dura sealing.	Minor case series/case reports with <20 patients.
	All evidence levels, including case reports, that including safety data were acceptable for safety analysis inclusion.	Case series where different surgical techniques (including technical notes) are compared rather than the potential effects of fibrin sealants.
		Cost data reports without efficacy or safety data
		Reviews, editorials, opinions, comments, and letters without original data.
		Non-clinical (ie, experimental, animal, or in vitro) studies.
		Clinical trials with major quality issues and a high risk of bias were excluded from efficacy analysis, but could be included in safety analyses.
Types of Participants	Patients (irrespective of age, sex or race) who: a) had undergone neurosurgical intervention of the brain or spine where fibrin sealants had been used to seal dura in order to treat acute CSF leaks and/or to prevent CSF leaks	Patients with spontaneous or trauma-related CSF leaks.
	or presented with persistent CSF leaks after neurosurgical procedures where conservative treatment had failed and where attempts to close the CSF leak using fibrin sealants had been done	Patients where fibrin sealant was used for haemostatic effect only.
	or had spinal tap procedures with persistent CSF leaks.	
Types of Interventions	Fibrin sealant either as liquid glue (drops or spray) or solid dry patch applied to: a) acute CSF leak after surgery as an add-on treatment covering the suture lines	Fibrin sealants mixed with other products such as bone chips/powder, hydroxyapatite etc.
	b) acute CSF leak from dural patch of autologous fascia, pericranium or collagen-based dura substitute to cover the suture lines and patch.	Comparisons of different fibrin sealant application methods.
	c) persistent CSF leak after neurosurgical procedures and spinal puncture	Interventions where fibrin sealant is not applied on or close to dura/dura grafts or suture lines involving dura to provide dura sealing.
		Reports on the sole use of autologous/homemade fibrin sealants (non-standardized product characteristics).
Types of comparators	Placebo (sham) or no fibrin sealant treatment (just sutures).	Studies where different patches/dura substitutes are compared and fibrin sealants are used as the standard of care.
	Medical treatment with acetazolamide.	
	Conservative treatment with persisting CSF leak	
Types of Efficacy Outcome Measures	Could include (but not limited to): 1. After Surgery: a) acute CSF leaks (preferably after Valsalva maneuver to increase intracranial pressure); b) Early (1 week) persistent CSF leaks (β2-transferrin, computed tomography or magnetic resonance imaging); or c) Late (>4 weeks) persistent CSF leaks (β2-transferrin, computed tomography or magnetic resonance imaging).	
	2. After CSF Leak: a) the number of patients with effective closure of the CSF leak	
Safety Outcome Measures	Could include (but not limited to):	
	Mortality.	
	Overall incidence of serious adverse events (quantitative).	
	Overall incidence of adverse events related to fibrin sealants (quantitative).	
	Qualitative assessment of specific adverse events/serious adverse events related to use of fibrin sealants.	
	Reoperation rate due to CSF leaks.	

**Table 3:** Defined Levels of Evidence in Literature Search Articles.

1a	Systematic reviews (with homogeneity) of randomized controlled trials
1a-	Systematic reviews of randomized trials displaying worrisome heterogeneity
1b	Individual randomized controlled trials (with narrow confidence interval)
1b-	Individual randomized controlled trials (with a wide confidence interval)
1c	All or none randomized controlled trials
2a	Systematic reviews (with homogeneity) of cohort studies
2a-	Systematic reviews of cohort studies displaying worrisome heterogeneity
2b	Individual cohort study or low quality randomized controlled trials (<80% follow-up)
2b-	Individual cohort study or low quality randomized controlled trials (<80% follow-up / wide confidence interval)
2c	“Outcomes” research; ecological studies
3a	Systematic reviews (with homogeneity) of case-control studies
3a-	Systematic reviews of case-control studies displaying worrisome heterogeneity
3b	Individual case-control study
4	Case-series (and poor quality cohort and case-control studies)
5	Expert opinion without explicit critical appraisal, or based on physiology, bench research or “first principles”

Based on data from the Oxford Centre for Evidence-based Medicine [28], which is available at: http://www.cebm.net/index.aspx?o=1025

Among the identified articles meeting the selection criteria, full versions were used for data analysis and a secondary search of the listed citations was performed to ensure that all relevant publications were included. English-language publications were the primary focus, although studies in Italian and German were also assessed for potential importance/relevance with those reporting adverse events being translated (if the English abstract was not sufficient or available) and included in the safety analysis.

### Data Appraisal and Extraction

The following data were extracted from each identified publication: trial characteristics including its design and outcome measures, setting, location of care, and country; participant descriptions including sample size, age, sex, disease, and surgical procedure; intervention information including the type and brand of fibrin sealant used and, if comparator used, the type and brand; and details of study efficacy and/or safety results.

## Results

### Characteristics of Included Studies

**Fig 1:**
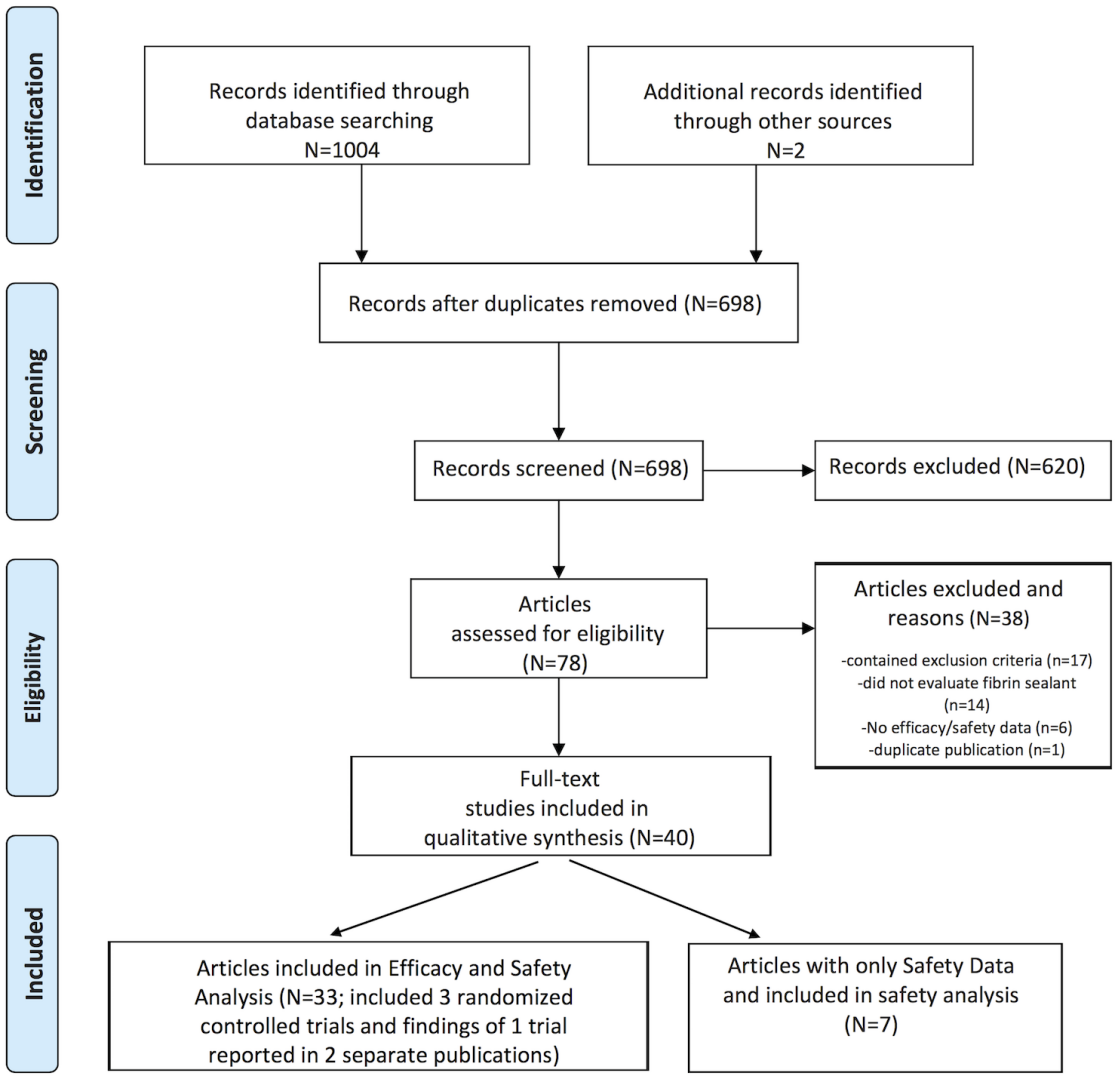
PRISMA Flow Diagram of the Selection Process to Identify Studies for Review.

The MEDLINE/PubMed and EMBASE literature searches identified a total of 1004 potential citations with 3 citations identified through the www.clinicaltrials.gov search: no citations were identified through the search of the Cochrane Library. Of the 3 citations identified through the clinicaltrials.gov site at the time of the literature review, results were posted for one study (NCT00681824 [29]), one study was listed as completed without posted results (NCT01355627 [30]); and a citation (NCT01174992 [31]) with results found in a European Medicines Agency report [24]. The flow chart of the literature search and the citation selection, review, and inclusion and exclusion process are illustrated in Fig 1.

After the application of methods to remove 309 duplicate references (Reference Manager Profession, Version 12, Thomson Reuters and manual screening), a total of 697 citations remained. The abstracts of these citations were screened for inclusion and exclusion criteria and relevance, with 620 of these excluded as they did not cover the subject of the literature review. The full-text of the remaining 78 papers were reviewed and, of these, 40 met the a priori defined selection criteria with 33 meeting that for efficacy and some of these reporting on safety. Two of the 33 papers reported on patients enrolled in a single trial [10, 11]. Seven papers contained specific safety reporting on fibrin sealant use in the predefined indication of neurosurgery for dura sealing. The citations were evaluated for their levels of evidence (Table 4) [28]. Three of the studies were randomized controlled trials of evidence levels 1b to 2 [24, 29, 32] with the remaining being retrospective case series or cohort studies of evidence levels 2b to 4.

**Table 4:** Literature Search: Summary of Levels of Evidence of the Included Studies.

Level of evidence [28]	Trial type	Originally selected studies	Studies included	Total number of patients in studies exposed to fibrin sealant
**I**	Meta-analysis	0	0	0
**Ib—Ib-**	Randomized controlled trial	2	2	123
**II**	Prospective cohort studies / randomized controlled trial with quality issues	2	2	126
**III**	Case-control studies	2	2	167
**IV**	Prospective and retrospective case series	27	26	2519
**Total**	**-**	33*	32*	**2935**

Note: multiple publications addressing data from the same study are counted once:

*The total of included studies for efficacy and safety is 32, two studies are reporting on the same patients [10, 11].

Among the reviewed citations, a total of 2935 participants were exposed to fibrin sealants. Details of the study designs and participants, interventions, comparisons, outcome measures, and authors’ conclusions are summarized for each trial in Table 5. By study type, 249 participants were exposed to liquid fibrin sealants in 2 of the 3 randomized controlled trials [24, 29]. A total of 126 participants were exposed to fibrin sealants in the remaining randomized controlled trial [32] and one prospective cohort study [33]. In 2 case controlled studies [34, 35], 167 participants were exposed fibrin sealants, with the remaining 25 case series analyses reporting on the remaining 2393 participants.

**Table 5:** Literature Search: Summary of Studies with Results on Efficacy and Safety.

Author, year	Study design	Intervention comparison	Population	Total number of patients	Patients exposed to fibrin sealant	Efficacy (primary endpoint in bold if stated in paper)	Safety	Author conclusions	Evidence level
**Treatment of acute (intraoperative) cerebrospinal fluid (CSF) leaks**									
Green, et al [24] / NCT 01174992 [31]	Randomized controlled trial	Fibrin sealant (Evicel) OR Sutures (Control) (2:1 random)	≥18 years, undergoing elective craniotomy or craniectomy for pathological processes in the posterior fossa or in the supratentorial region and who were demonstrated to have persistent CSF leakage following primary attempt at suture closure of the dural incision	139 (89:50)	89	**Intraoperative watertight dura closure** was observed in a greater proportion of fibrin sealant-treated than control group subjects (92.1% [82/89] versus 38.0% [19/50] of subjects respectively, p<0.001)	Post-operative CSF leakage after treatment occurred in 6.7% (6/89) of fibrin sealant and 2.0% ((1/50) of control group patients. One subject in each group developed meningitis The incidence of adverse events was comparable: 64.0% in fibrin sealant group versus 62.0% in control group	Superiority of fibrin sealant over sutures in establishing intraoperative watertight closure of the dural incision was demonstrated. Post-operative CSF leaks was slightly higher in the fibrin sealant group. General safety (adverse events) was comparable	1b
Hobbs 2011 [36]	Retrospective case series	If intraoperative CSF leak: fibrin sealant (Tisseel) + Spongostan + fibrin sealant + Spongostan followed by packing of the sphenoid sinus. No comparison	Patients undergoing pituitary surgery procedures (96 primary procedures and 24 revisions) with intraoperative CSF leaks	120	28	28/120 intra operative CSF leaks were treated with the intervention. All intra operative leaks were managed using the fibrin sealant and gelatin sponge technique. One patient (3.6%) had a CSF leak that continued post-operatively that required a post-operative ventriculoperitoneal shunt with the CSF leak subsequently resolving	One case of aseptic meningitis	Simple conservative technique with low incidence of postoperative CSF leak	4
**Prevention of postoperative CSF leaks**									
clinicaltrials.govNCT 00681824 [29]	Randomized Controlled Pilot study	Fibrin sealant (Tisseel) plus Standard of care OR Control (Standard of Control only) closure of dura defect with suture + patch (autologous fascia, pericranium or collagen based dura substitute)	Age ≥3 years, undergoing elective craniotomy / craniectomy for pathological processes in the posterior fossa that resulted in dura defects requiring dura substitution for closure	62 with additional 13 "run in" patients	34	**Incidence of CSF leakage observed 33 days after surgery (computed tomography/magnetic resonance imaging/clinical)** was 78% in the fibrin sealant group compared with 74% in the control group (p = 0.72). Surgical revisions occurred in 3 vs. 0%, and surgical site infection in 2 vs. 0% (fibrin sealant vs. control, respectively)	Serious adverse events were reported in 21% vs. 14% and other adverse events in 70 vs. 71% (fibrin sealant vs. control, respectively)	Fibrin sealant was not found to be superior in preventing CSF leakage postsurgery when compared with suture/patch. Safety, albeit not detailed, seemed comparable in the 2 groups	1b-
Nakamura 2005 [32]	Randomized controlled trial	Group 1: Suture + Goretex; Group 2: Regimen 1 + fibrin sealant autologous; Group 3: Regimen 1 + fibrin sealant (Bolheal)	Patients with spinal cord tumors and related illnesses undergoing spinal surgery	39	26 (13 autologous fibrin sealant; 13 fibrin sealant)	Primary endpoint was drain output with no statistical difference between autologous fibrin sealant and fibrin sealant. No postsurgical CSF leak was observed in any of the patients in the 3 groups	No complications observed and no adverse events reported	No definitive CSF leaks were observed in any group	2b
Than 2008 [33]	Prospective cohort (polyethylene glycol) Retrospective control (fibrin sealant)	Polyethylene glycol versus fibrin sealant	Patients undergoing cranial posterior fossa surgery	200	100	In the PEG group, two of 100 (2%) patients developed CSF leak postoperatively compared with 10 of 100 (10%) patients in fibrin sealant group (p = 0.03)	There were no significant differences in the rates of pseudo-meningocele, meningitis, or other postoperative interventions	Polyethylene glycol dural sealant to the closed dural edges may be effective at reducing incisional CSF leak after posterior fossa surgery	2b-
Tamasauskas 2008 [34]	Case control study	Method 1: Packing the sella and sphenoidal sinus: autologous fat and restoring the bone defect of the sella with autologous bone OR Method 2 (fibrin sealant): Multilayer sella closing using oxidized cellulose (Surgicel) and fibrin sealant based collagen fleece (Tachosil)	Patients undergoing trans-sphenoidal operations for pituitary adenoma that developed intraoperative CSF leak	313 (58 with intra-operative CSF leak)	29	3/29 (10%) developed postoperative CSF leak with Method 1 compared with 0 with Method 2 (fibrin sealant)	Hypopituitarism (1 versus 0), Sphenoidal sinusitis (2 versus 0), Permanent diabetes insipidus (2 versus 1), Paresis n. oculomotorius (1 versus 0), and Intraventricular haemorrhage (0 versus 1) when comparing number of cases with Method 1 versus Method 2 (fibrin sealant)	Multilayer sella closing using oxidized cellulose (Surgicel) and fibrin sealant based collagen fleece appeared to be the most reliable one, as no postoperative CSF leakage applying this technique was observed	3b
Yoshimoto 1997 [35]	Case control study	Case: Suture + fibrin sealant (Bolheal / Veriplast P). Control: Suture	Patients mean age of 60 years undergoing craniotomy for unruptured aneurysm	183	138	**CT verified fluid collection** occurred in 19/45 patients (42%) without fibrin sealant and in 36/138 patients (26%) with fibrin sealant, (p<0.05). All fluid collections detected were transient and no patient required second surgery due to CSF leak	No infections, including meningitis and no other adverse effects were detected	Fibrin sealant is a useful surgical tool for the prevention of postoperative extradural fluid collection through the dural sutures	3b
Cappabianca 2004 [37]	Retrospective cohorts	Group 1: Fibrin sealant (Tissucol); Group 2: Collagen fleece; Group 3: Fibrin sealant + Collagen fleece	Patients undergoing sellar repair after endoscopic endonasal transsphenoidal surgery.	242	29	Group 1 (fibrin sealant, n = 16): CSF leaks occurred in 2/16 (12.5%) patients and 6/16 patients required a lumbar drainage. Group 2 (collagen fleece, n = 6)): no case of CSF leak occurred, and 1 patient required a lumbar drainage. Group 3 (fibrin sealant + collagen fleece, n = 13) No CSF leak occurred in the postoperative course and no case required a lumbar drainage	No safety data reported	These data seem to confirm the synergistic action of the 2 materials (fibrin sealant + collagen fleece in assuring a safer and effective sellar repair in case of an intraoperative CSF leak	4
Seda 2006 [38]	Case series	Group 1 (no intraoperative CSF leak): Surgicel. Group 2 (intraoperative CSF leak): Surgicel + fibrin sealant (Beriplast)	Patients undergoing reconstructing the sellar floor after transsphenoidal procedures	567	64	Group 1: No delayed CSF leak, meningitis, or visual loss. Group 2: 1/63 (1.6%) disclosed a delayed postoperative CSF leak treated by reoperation	Group 1: No meningitis, or visual loss. Group 2: 1/64 patient developed meningitis but no overt CSF leak, treated by antibiotics	Sellar floor closure with Surgicel and fibrin sealant without grafting/use of implants is a safe and efficient method to prevent postoperative complications after trans-sphenoidal procedures	4
Esposito 2008 [10]; Esposito 2013 [11]	Prospective case series	Collagen based dural replacement covered with fibrin sealant (Tisseel) No comparison	Patients undergoing a variety of neurosurgical procedures (cranial, transsphenoidal, spinal) and requiring a dural graft implant. In a later publication 111 patients underwent follow up evaluation 5 years after surgery [11]	208 (111 at 5-year follow-up)	208	1/208 patients (0.5%) experienced postoperative CSF. Postoperative magnetic resonance imaging showed signs of moderate inflammatory response in only one patient, who did not present any postoperative clinical symptom nor neurological deficits	In 3 patients undergoing reoperation the dural substitute appeared to have promoted a satisfactory dural regeneration (histology). No or minimal adherences with the other tissues and the brain cortex. At 5 years follow up, 5 patients (4.5%) had undergone reoperation and 2 out of 5 experienced subcutaneous fluid collections	The collagen- only biomatrix is a safe and effective dural substitute for routine neurosurgical procedures used together with fibrin sealant. Data are supported by a 5 year follow survey 111 patients	4
Cappabianca 2006 [7]	Case series	Collagen foil + fibrin sealant (Tisseel). No comparison	Patients undergoing endoscopic endonasal transsphenoidal surgery for a variety of pituitary lesions. Patients with intraoperative CSF leaks or who needed sella floor reconstruction received the intervention (n = 15)	72	15	15/72 patients (20.8%) required implant of the collagen foil. 9/15 (60%) had intraoperative CSF leak. 1/15 (6.7%) developed postoperative CSF leak	1/15 (6.7%) presented with meningitis. The patient required a reoperation for CSF fistula repair and intravenous antibiotics. No other adverse events observed	This experience suggests that the use of Collagen foil + fibrin sealant in trans-sphenoidal surgery is safe and biocompatible	4
Parlato 2011 [39]	Case series	Overlay with collagen foil + fibrin sealant (no dural sutures). No comparison	Patients undergoing cranial and spinal procedures with the need of dural reconstruction	74	74	Clinical and neuroradiological (magnetic resonance scan at 1 week, 1 month and 1 year) findings were normal. No graft rejections or CSF leak were observed	At 12 months follow-up, no local toxicity or complications such as CSF leaks, adherences or inflammation were observed. No further adverse events reported	Following dural reconstructions with collagen foil + fibrin sealant without surgical sutures, no local toxicity or complications were observed for up to 1 year	4
Gazzeri 2009 [40]	Prospective case series	Collagen biomatrix + fibrin sealant (Tissucol); No comparison	Patients >18 years of age with cranial or spinal dural defect requiring placement of a dural substitute with a life expectancy >6 months. Exclusion criteria: internal or external CSF shunt, known or suspected systemic or local infection, known systemic collagen disease, usage of corticosteroids, previous radiotherapy or chemotherapy	60	60	56 patients had cranial surgery, 4 had a spinal operation. At 7-days follow-up, 2/60 (3.3%) had CSF leak. Neither patient needed reoperation. A subgaleal fluid collection in two patients resolved after tapping. Of the 56 patients who reached the 3-month follow-up, none had a CSF leak	Of the 56 patients who reached the 3-month follow-up, none developed meningitis, wound infection or CSF fistulae. No other adverse events reported	The use of fibrin sealant reduces suturing and facilitates the implantation of the collagen biomatrix	4
Murai 2013 [41]	Retrospective case series	Sutures + Gelfoam + fibrin sealant. No comparison	Fifty-one consecutive patients (age 15–71) undergoing bifrontal craniotomy with frontal sinus exposure for frontal base lesions. Patients without exposure of bony frontal sinus or the mucous membranes of frontal sinus were excluded	51	51	Postoperative CSF leakage: 0 with a postoperative follow-up period of 1–84 months (mean: 36.8 months)	No meningitis and no other adverse events reported during follow up	The use of Sutures + Gelfoam + fibrin sealant indicates effectiveness in the prevention of frontal sinus related postoperative complications	4
Cappabianca 2010 [42]	Case series	Fibrin sealant (Tisseel) inside tumor cavity to fill dead space + other materials. No comparison	40 subjects undergoing endoscopic endonasal approach for different sellar and skull base lesions in which an intraoperative CSF leakage was evident	40	40	No postoperative CSF leaks	No adverse events reported	The injection of fibrin sealant proved to be effective in filling/sealing postoperative “dead spaces” and treating minor or initial CSF leaks	4
Van Velthoven 1991 [43]	Case series	Septal bone + fibrin sealant (Beriplast / Tissucol) + Spongycel for reconstruction of the sellar floor and sphenoid sinus. No comparison	Patients undergoing transsphenoidal (sublabial, transseptal) operations due to a variety of sellar pathologies	119	119	Overall incidence of postoperative rhinorrhea was 1.6%. Intraoperative CSF leakage occurred in 15/119 (12.6%) of cases. Postoperative rhinorrhea occurred and persisted in 2 of the 15 patients with intraoperative CSF leakage. None of the 104 patients without intra- operative CSF leakage developed postoperative rhinorrhea	One patient suffered postoperative meningitis, associated with intra-operative and postoperative CSF leak treated with antibiotics, no sequelae. One patient developed hepatitis A—unrelated to the use of fibrin sealant	This supports the view that sellar and sphenoidal sealing with fibrin sealant instead of muscle or fat tissue does not raise the incidence of post-operative rhinorrhea	4
Yin 2005 [44]	Retrospective case series	4 techniques were evaluated: 1. Gelatin foam; 2. Gelatin foam + fibrin sealant; 3. Gelatin foam + fibrin sealant + autologous fat; 4. Regimen 1, 2, or 3 + CSF drainage	Consecutive patients age 13–72, undergoing sellar floor reconstruction following transsphenoidal surgery	176	77	Patients developed postoperative CSF leaks in 0, 0, 1.6%, and in 0% of the cases (tech. 1,2,3,4, respectively). Magnetic resonance imaging follow-up after 12 months showed progressive resorption of material in sella	No postoperative complications such as CSF rhinorrhea, allergic rhinitis, meningitis, or pneumocranium and no deaths	Gelatin foam and fibrin sealant in cranial reconstruction is safe and effective in preventing postoperative complications following trans-sphenoidal surgery	4
Jankowitz 2009 [45]	Retrospective case series	Suture + fibrin sealant (Tisseel) versus suture alone	Patients undergoing lumbar spine surgery experiencing an incidental durotomy	4835	278	Incidental durotomy occurred with an overall incidence of 11.3%. Fibrin sealant was used during 278 of these cases (50.8%) to augment the dural closure. 31/269 (11.5%) without fibrin sealant developed CSF leak vs. 33/278 (11.9%) with fibrin sealant (not significant). Logistic models evaluating age, sex, redo surgery, and the use of fibrin sealant revealed that prior lumbar spinal surgery was the only univariate predictor of persistent CSF leak, conferring a 2.8-fold increase in risk	There were no complications associated with the use of fibrin sealant	In patients who experienced an incidental durotomy during lumbar spine surgery, the use of fibrin sealant for dural repair did not significantly decrease the incidence of a persistent CSF leak	4
Kassam 2003 [46]	Retrospective case control (historical) study	Suture + fibrin sealant (Tisseel) versus suture	Patients undergoing anterior cranial base, infratemporal, and retromastoid surgical procedures	253	72	10/181 (5.5%) control patients versus 0% fibrin sealant treated patients developed postsurgical CSF leaks (p = 0.067). In patients undergoing anterior cranial base procedure, CSF leaks occurred in 16 versus 0%, (control versus fibrin sealant)	1.1% of controls versus 0 developed pneumocranium (control versus fibrin sealant, respectively). No further adverse event data available	Fibrin sealant reduces the incidence of postoperative CSF leaks and tension pneumocranium	4
Kurschel 2007 [47]	Retrospective case series	Mixture of fibrin sealant (Beriplast) and Surgicel	Endoscopic third ventriculostomies were performed in 20 hydrocephalic children with a mean age of 22 months	20	20	One child developed an asymptomatic CSF leak that was managed conservatively. The leak was attributed to the age of the child and assumed poor CSF absorption ability	No adverse effects regarding the material used for sealing were observed over a mean follow-up of 23 months	Fibrin sealant and Surgicel seems to be safe, and this technique effectively in reducing the risk of CSF leaks in this patient population	4
Parker 2011 [48]	Retrospective case series	Sutures + different graft types (cadaveric pericardium, Durepair, and EnDura) augmented with either: 1. No sealant; 2. Fibrin sealant (Tisseel); 3. Duraseal (PEG matrix)	Consecutive patients ≤18 years old undergoing primary Chiari malformation Type I decompression using duraplasty	114	75	Fibrin sealant was used in 75 patients, DuraSeal in 12, and no tissue sealant was used in 27 patients. No efficacy data presented	The overall complication rate was 21.1% (aseptic meningitis, pseudo-meningocele, or a CSF leak requiring reoperation). Complication rates for tissue sealants were 14.8% for no sealant, 18.7% for fibrin sealant, and 50% for DuraSeal (p<0.05). A subgroup treated with Durepair and DuraSeal had a 56% complication rate. Cases of aseptic meningitis were linked to one graft (Durepair)	The use of tissue sealants to augment duraplasty may not provide any additional benefit	4
Gazzeri 2011 [49]	Case series	Oxidized cellulose for dura defect + fibrin sealant (Tissucol). No comparison	Patients either scheduled (n = 21) or emergency (n = 24) undergoing supratentorial craniotomies with dural defects ranging from 10–40 mm (min-max)	467	45	Postoperatively, 3/45 (6.7%) developed subgaleal fluid collection, which resolved conservatively in 2 cases	There were no other complications or reoperations	Oxidized cellulose + fibrin sealant, is a sutureless, fast, and valid alternative to small dural defect closure methods	4
Gillman 1995 [50]	Retrospective case series	Fibrin sealant (Tisseel) indural closure in translabyrinthine resection. No comparison	Patient undergoing surgical and non-surgical treatment for acoustic neuroma	83	52	Postoperative CSF leaks in 6/52 (11.5%) of patients treated with fibrin sealant undergoing translabyrinthine resection	No safety data reported	Further randomized controlled trials to evaluated fibrin sealant for CSF leaks are merited	4
Hida 2006 [51]	Prospective case series	Polyglycoic acid (PGA) sheet + fibrin sealant (Bolheal). No comparison	Patients undergoing spinal surgery requiring intraoperative dura repair	160	160	Postoperative subcutaneous CSF accumulation occurred in 10/160 (6.3%) cases	No complications such as allergic reaction, adhesion, or infection	The polyglycoic acid-fibrin sealant sheet is a viable alternative method for dural repair in spinal surgery	4
Reddy 2002 [52]	Retrospective case series	Fibrinogen / thrombin based collagen fleece (Tachocomb). No comparison	Consecutive patients undergoing neurosurgical and spinal procedures (intracranial tumors, cerebellar tumors, traumatic lesions, spinal lesions, microvascular decompression for trigeminal neuralgia, vascular diseases, infections)All patients, in whom a primary dural closure or a watertight closure was not possible and in whom autograft harvest was either impractical or impossible were considered eligible	288	288	Postoperative CSF leaks developed in 5/288 (1.7%) patients, requiring reoperation. Rebleeding observed in 1 patient. In 4/288 (1.4%) patients, there was notable subcutaneous CSF accumulation without CSF-leak requiring lumbar drainage	No superficial or deep wound infections or aseptic meningitis were noted. No other adverse events reported	Fibrin sealant based-collagen fleece is an adequate alternative for dural substitution: it is safe, watertight and efficient	4
Reddy 2003 [53]	Retrospective case series	Fibrin sealant based collagen fleece (Tachocomb). No comparison	421 brain surgery cases, 42 of which involved the skull base	421	421	12/421 (2.8%) developed postoperative subcutaneous CSF leak. 3 patients required reoperation	No safety data reported.	Fibrin sealant based collagen fleece is watertight and effective	4
Nistor 1997 [54]	Case series	Fibrin sealant based collagen fleece (Tachocomb). No comparison	Patients undergoing neurosurgical intervention for primary skull base pathology	44	44	0 postoperative CSF leaks with a mean follow up time of 18 months	No cases of meningitis during a median follow-up period of 18 monthsPostoperative magnetic resonance imaging did not reveal any CSF or infectious abnormalities. One case of pneumocephalus	Experience from this case series shows good sealing performance of fibrin sealant based collagen fleece used in skull base surgery	4
Cho 2011 [55]	Retrospective case series	Intraoperative CSF leaks repaired with fibrin sealant-based collagen fleece (TachoComb). No comparison	Patients undergoing transsphenoidal surgery for pituitary adenoma (Hardy grade I-IV) experiencing intraoperative CSF leak	307	90	2/90 (2.2%) patients developed postsurgical CSF leak (rhinorrhea)	No hypersensitivity reactions against fibrin sealant and no infections within 16 months of follow-up (magnetic resonance imaging)	This technique is an alternative method to the traditional autologous tissue graft technique	4
Black 2002 [56]	Retrospective case series	Fat + fibrin sealant + Gelfoam/ Surgicel applied to spinal dural tear. No comparison	Patients undergoing spinal surgery for various pathologies where unintended spinal tears occur	1650	27	Of 27/1650 unintended dural tears, 1/27 (3.7%) developed postoperative CSF leak treated with skin suture	No safety events reported	The use of a fat graft is recommended as a rapid, effective means of prevention and repair of CSF leaks following spinal surgery	4
Weber 1996 [57]	Retrospective survey	Endonasal duraplasty, external duraplasty (frontoorbital or transfrontal extradural approach) by underlay/onlay technique. Fibrin sealant (Tisseel) used to seal grafts of mucosal flaps. No comparison	Consecutive sample of patients undergoing duraplasty for repair of a dural lesion that occurred as a complication of endonasal sinus surgery	47	47	42 patients were followed up 5 years after surgery (range: 6 month to 15 years). Fluorescein test, performed in 43% (20/47) of the patients was negative in all case. Duraplasty was clinically intact in 100%	26% of the patients had had 1 or more episodes of bacterial sinusitis. No cases of CSF rhinorrhea or meningitis	Allogeneic connective tissue in combination with fibrin sealant has proved suitable as a graft material	4
**Treatment of persisting CSF leaks**									
Cassano 2009 [58]	Retrospective case series	Overlay apposition of a lower turbinate mucoperiostal graft fixated with fibrin sealant and Surgicel. No comparison	Adults undergoing anterior skull base repair of persisting CSF fistulae treated endoscopically using overlay apposition of graft + fibrin sealant + Surgicel	125	125	The success rate at first attempt was 94.4% There were 7/125 (5.6%) cases of postoperative recurrent CSF leakage (all resolved at 3 month follow-up)	No safety data reported	Repair of anterior skull base CSF fistulae with the described technique with fixators (fibrin sealant) and supports (Surgicel, Spongostan), permits the restoration of dural continuity in a majority of cases	4
Cappabianca 2010 [42]*	Case series	Fibrin sealant (Tisseel) locally injected with application system/Tuohy needle every 48 hours until CSF leak treated. No comparison	10 subjects with postoperative CSF leakage after transsphenoidal, spinal, posterior fossa and transcortical transventricular tumour removal surgery	10	10	All CSF leaks or collections were closed after 1 to 5 applications of fibrin sealant. Successful results were stable with a follow-up ranging from 6 months to 3 years	No adverse events reported	The injection of fibrin sealant may add another possibility in the treatment of post-operative CSF leaks	4

*These 10 patients were a subpopulation among the 50 patients in the Cappabianca case series publication [42].

Note: Fibrin sealant brand name included if cited in publication.

CSF = cerebrospinal fluid

Notably, one case series citation by Cappabianca and colleagues of 50 subjects exposed to fibrin sealant, 40 were evaluated for postoperative CSF leak efficacy information: 10 subjects were evaluated for treatment of persisting CSF leak efficacy [42]. Although the 10 subject evaluation did not meet our literature search criteria, the information from this case series subpopulation was included for completeness [42].

### Fibrin Sealant Efficacy and Safety in Randomized Controlled Trials

As detailed in Table 6, the quality of each of the 3 randomized controlled trials [24, 29, 32] varied in their level of evidence. The largest trial by Green and colleagues was conducted under the regulatory requirements for obtaining an indication in the European Union and was presented with an overall high study quality [24]. The remaining 2 randomized controlled trials had small numbers of participants [29, 32]. The one trial posted on clinicaltrials.gov (NCT00681824 [29]), a pilot study to investigate the efficacy and safety of fibrin sealant (Tisseel) for use in posterior fossa surgery as an adjunct to dura and dura substitute sutures in preventing postoperative CSF leakage, was performed without any formal sample size calculation. Limitations to the trial by Nakamura and colleagues [32] include its limited number of spinal patients with an *a priori* sample size calculation and use of a pseudo randomization (clinical chart number system), which may have resulted in the trial being underpowered and having bias. These design and power limitations limit the ability to draw conclusions of efficacy from their findings [32].

**Table 6:** Appraisal of Study Quality for Three Randomized Controlled Trials*.

Study	Primary outcome stated?	Inclusion / exclusion specified	^†^Generation of allocation sequence adequate?	^†^Allocation concealment adequate?	a priori sample size/power calculation	Blinded outcome assessors?	Blinded patients?	Consecutive cases?	Intent-to-treat analysis	Lost to follow up (%)
Green 2014 [24]	Yes	Yes	Unclear	Unclear	Yes	No	Yes	Yes	Yes	(1–2%)
Clinicaltrials.gov NCT 00681824 [29]	Yes	Yes	Unclear	Unclear	No	Yes	Yes	Yes	Yes	0
Nakamura 2005 [32]	Yes	Yes	No	No	Yes^#1^	No	Unclear	Yes	No	0

Possible answers for each section are: Yes (low risk of bias [ROB]), No (high ROB) and Unclear (Uncertain ROB).

Unclear denotes where there is insufficient information in the publication to permit a clear judgment).

ITT = intent to treat.

*Inspired by the risk of bias table (Figure 8.6.a) in Higgins JPT, Altman DG (Eds). Chapter 8: Assessing risk of bias in included studies. In: Higgins JPT, Green S (Eds). *Cochrane Handbook for Systematic Reviews of Interventions*. The Cochrane Collaboration, 2008. Availablwww.cochrane-handbook.org [59].

^**†**^The minimum criteria for adequate concealment are based on data from Schultz and Grimes, 2002 [60].

^#1^: Sample size done, reported but not followed in actual study.

### Efficacy and Safety of Fibrin Sealants, by Indication

The following subsections describe the strength of the overall evidence for the efficacy and safety of fibrin sealants in the prevention and/or treatment of acute or persistent CSF leaks, by these indications.

#### Treatment of acute (intraoperative) CSF leaks

One of the 3 randomized controlled trials was designed to evaluate the efficacy and safety of a liquid fibrin sealant containing both thrombin and fibrinogen as an adjunct to dura sutures in patients undergoing elective cranial surgery (supratentorial/posterior fossa) who experienced a CSF leak after primary suture closure of the dura mater [24]. This clinical trial [24], which was identified from the clinicaltrials.gov website, was used to submit data to the European Medicines Agency (EMA) to obtain the indication of suture line sealing in dura mater closure. Among the 139 patients evaluated, fibrin sealant was used as an adjunct to sutures in 89 patients with the remaining “control group” participants managed through the use of additional sutures. The primary endpoint was the percentage of patients attaining intraoperative watertight dura closure. This endpoint was met in 92.1% of fibrin sealant managed patients and 38% of those managed with additional sutures (p<0.001). This significant difference in intraoperative CSF leaks had no effect on the frequency of postoperative leaks, which occurred in 6.7% of fibrin sealant patients and 2% of the additional suture control group. Adverse event incidence rates were similar between the groups and there was no increased occurrence of safety events in those exposed to fibrin sealant. The findings of this Evidence Level 1b, well-designed, randomized controlled trial supports the efficacy and the safety of fibrin sealants over additional sutures in attaining intraoperative dura sealing.

In an Evidence Level 4, retrospective case series of 120 patients undergoing pituitary surgery, fibrin sealant was used with different materials to pack the sphenoid sinus [22]. The intraoperative leak rate was 3.6% and a single case of aseptic meningitis was reported. Limitations in the study design preclude firm efficacy and safety conclusions from this analysis.

#### Prevention of Postoperative CSF leaks

A randomized controlled pilot trial identified only on the clinicaltrials.gov website provided Evidence Level 1b on the use of liquid fibrin sealant in the prevention of postoperative CSF leaks in patients undergoing cranial surgery [29]. In the 62 patients enrolled, the dura mater was closed with sutures and patches (standard of care) alone or with liquid fibrin sealant used as an adjunct. The primary endpoint, the prevention of postoperative CSF leaks, was compared between those treated with standard of care alone and those who received fibrin sealant adjunct management. The endpoint was evaluated clinically and with extensive computed tomography and magnetic resonance imaging techniques (CT/MRI) at 33 days and 5 weeks postoperatively. At the first postoperative evaluation (33 days), the percentage of patients with postoperative CSF leaks was similar between the groups (78% in fibrin sealant adjunct and 74% in standard of care alone group). Safety events, including surgical revisions and infections, were distributed evenly in the two groups. The findings of this trial do not establish the prevention of CSF leaks with fibrin sealants, but did report a comparable safety profile between the sutures and patches standard of care and the use of fibrin sealant as an adjunct.

A smaller (39 patients) randomized controlled trial provided Evidence Level 2b on the efficacy of liquid fibrin sealant adjunct to sutures and Goretex in the closure of spinal cord dura [32]. As mentioned previously, this study by Nakamura and colleagues had several design-related quality issues that limit the interpretation of their findings such as use of a pseudo randomization system, an a priori sample size, and the potential for being underpowered and biased [32]. In the study, postoperative CSF leakage was the secondary endpoint with the primary endpoint being haemostasis. CSF leakage postoperatively was not observed in any of the study patients and no adverse events or complications were reported. Although not statistically significant, the data demonstrates that fibrin sealants were effective in preventing postoperative CSF leakage in the patients evaluated.

Three studies used a case control design [33–35] to assess fibrin sealants in preventing CSF leaks. In a study of 200 patients undergoing fossa posterior surgery with intraoperative CSF leaks, Than and colleagues [33] compared the use of fibrin sealants with PEG sealant. Overall, 10% of those exposed to fibrin sealants and 2% of those in the PEG group developed a postoperative CSF (p = 0.03). Yoshimoto and colleagues [35] compared treatment with a liquid fibrin sealant with sutures versus sutures alone (control group) in preventing postoperative CSF leaks in 183 patients undergoing craniotomy. The incidence of postoperative CSF leaks, verified by computed tomography, was 26% in the fibrin sealant group and 42% in the control group (p>0.05). Tamasauskas and colleagues [34] also used a case controlled approach to evaluate the incidence of postoperative CSF leaks in patients undergoing transsphenoidal surgery for pituitary adenoma with intraoperative detected CSF leaks. Intraoperative CSF leaks were treated with packing of the sella and sphenoidal sinus with different materials with a dry patch fibrin sealant or without (control group). No fibrin sealant-managed patient and 10% of those not managed with fibrin sealant developed a postoperative CSF leak. In these three case controlled trials [33–35], safety was reported to occur with a similar distribution between the fibrin sealant and control groups. In such trials where there is a high risk of bias that may influence the interpretation of efficacy and safety results, no consistency was shown of fibrin sealant efficacy and no safety issues were identified (Evidence level 3).

The majority of the selected papers reported data from case series that were either prospective or retrospective. In total, 22 such papers were selected with 2 papers reporting follow-up on same patient cohort [10, 11]. In these analyses, fibrin sealants were used predominantly in cranial procedures [10, 11, 39, 40, 41, 46, 48, 49, 52, 54] with low incidences (0 to 6.7%) of postoperative CSF leaks reported. In one case series where fibrin sealant was used in a subgroup of patients undergoing surgical treatment for acoustic neuroma with translabyrinthine resection, 11.5% of patients experienced postoperative CSF leak [50]. Fibrin sealant was reported to be effective, with postoperative CSF leaks rates of 0 to 6.7%, in patients undergoing transsphenoidal surgery [7, 37, 38, 42, 43, 44, 55, 57].

The use of fibrin sealant was assessed in 2 papers in patients undergoing transsphenoidal surgery. In the retrospective analysis by Cappabianca and colleagues [37], 2 of 16 patients (12.5%) treated with fibrin sealant alone presented with a postoperative CSF leak compared to none of the patients who received a fibrin sealant/collagen fleece combination. In the consecutive patient analysis by Yin and colleagues [44], the 77 patients who developed a visible intraoperative CSF leak were repaired with fibrin glue plus gelatin foam (n = 62) or with autologous fat graft and sellar floor reconstruction (n = 15), with the authors concluding that the use of fibrin glue with gelatin foam was effective and safe in preventing postoperative complications following transsphenoidal surgery.

In a small case series of 20 children with hydrocephalus where fibrin sealant was used, one child (5%)) developed a CSF leak [47]. In three case series of patients undergoing spinal surgery with dural repair where fibrin sealant was used to enforce the dural suture line, between 3.7% to 11.9% of patients developed postoperative CSF leak [45, 51, 56]. In general, in these case series, safety events occurred infrequently (meningitis, pseudomeningocele, pneumocephalus, see Table 5 for details). The results from these case series are limited in their interpretation on safety and efficacy due to design (Evidence level 4).

In two retrospective case series an attempt was made to compare the effect of fibrin sealant to a control in the incidence of persistent CSF leakage [45, 48]. In the analysis by Jankowitz [45] of 547 patients undergoing lumbar surgery and experiencing incidental durotomy during the first 3-month postoperative period, a total of 64 patients (11.7%) experienced a persistent CSF leak. There was no difference in the percentage of patients who experienced a persistent CSF leak in which fibrin glue was used to augment the dural closure (11.9%, n = 33) and those in which fibrin glue was not used (11.5%, n = 31) [45]. In a study by Parker and colleagues [48] of 114 consecutive children undergoing primary Chiari I malformation Type I decompression using duraplasty, the complication rate for tissue sealants was 14.8% in the 27 patients managed with no sealant and 18.7% for the 75 patients managed with fibrin sealant. The findings of these two studies [45, 48] indicate that there is no additional benefit of fibrin sealant use over other procedures; however, the retrospective case series design limits the ability to draw firm conclusions from these data.

#### Treatment of Persisting CSF leaks

Two case series reported the experience of treating patients with persisting CSF leaks after a variety of surgical intervention types (skull base, spinal, posterior fossa and other) [42, 58]. In the larger series of 125 patients by Cassano and colleagues [58], fibrin sealant used with turbinate grafts and Surgicel resulted in a postoperative recurrent CSF leakage rate of 5.6%. In the smaller case series of 10 patients who underwent different neurosurgical procedures and developed postoperative CSF fistulas or collection, the local injection of fibrin sealant was effective in treating the complication [42]. Among the 10 patients, 4 had undergone transsphenoidal surgery, 2 had undergone spinal surgery, 3 underwent posterior fossa surgeries and one underwent transcortical transventricular tumour removal. In this study by Cappabianca 2010 and colleagues [42], the liquid fibrin sealant applications were repeated every 48 hours until the disappearance of the leak. The number of applications ranged from one to five, with the application being successful in all cases. No adverse events were reported in these studies. The results from these case series are limited in their interpretation on safety and efficacy due to the reported design (Evidence level 4).

#### Results for Safety Evaluation Based on Specific Reported Adverse Events

As summarized in Table 5, few or no adverse events were reported in most of the studies. In the three randomized controlled trials where there is a specific comparison of treatment with and without fibrin sealants [24, 29, 32], no increased adverse events due to fibrin sealants was detected (Table 5).

Table 7 presents an overview of specific adverse events reported in publications that reported on the safety of fibrin sealants used in or near dura mater. From 1987, when the first safety case was reported, until 2014 there have been a total of 8 cases in neurosurgery [61–67]. These 8 cases included 2 allergic reactions [61, 65], 3 cases of aseptic meningitis [66, 67], one case of meningitis with fatal outcome with only limited information provided in the paper [62], one case of suspected air embolism with the use of a spray device [63], and one case of obstruction of epidural drain [64]. One of the 2 allergic reactions was supported by the finding of serologic tests (specific IgE and IgG to the allergen) [61]. What is unknown is whether this allergic reaction would have been observed if the product was applied topically, rather than systemic. The second allergic reaction, like most reported reactions was not confirmed as directly related to the product, but could be possibly related [61].

**Table 7:** Literature Search: Specific Reported Adverse Events, in Alphabetical order by first author.

Author, year	Report type	Adverse event reported and incidence	Comments
Beierlein, 2000 [61]	Case report	48 year old woman developed a liquid fistula after cranial surgery for cerebral metastasis. 29 days postsurgery, 4 ml fibrin sealant (Tissucol) was injected into subgaleal cavity. 40 days postsurgery another 4 ml of fibrin sealant was injected followed by clinical signs of **anaphylactic shock**. Serology identified aprotinin specific IgE and IgG.	Serology confirmed. Aprotinin is a bovine protein and as such is a potential allergen.
Czepko, 2006 [62]	Single case report	*Limited information from abstract in English*: 259 consecutive cases performed using a transsphenoidal approach. Intraoperative rhinorrhea occurred in 40 cases where fibrin sealant was used in sella reconstruction. Twenty-six (26) cases (group I) received Surgicel, artificial dura or fascia and fibrin sealant (Tissucol) and 14 cases (group II) received TachoComb + fibrin sealant, fascia or artificial dura. One patient died due to the meningitis (group I). No further information is available and no causality is given.	Very limited information and no causality to fibrin sealant given.
Felema, 2013 [63]	Case report	A 5-month old, Ex-33-week premature, 6.6-kg male scheduled for endoscopic cranial vault remodeling for sagittal craniosynostosis. At the completion of the surgical procedure and prior to skin closure, 4 ml of fibrin sealant (Tisseel) was applied for haemostasis at an approximate distance of 5 cm from the anterior endoscopic entrance site using an aerosolized spray applicator device (Easyspray) with nitrogen as a propellent gas at a pressure of 15 psi. Immediately after fibrin sealant delivery, a sudden drop in blood pressure from 88/42 to 38/21 was noted lasting 5 min. with no perceived change in blood loss. It was hypothesized that **air was introduced to the vascular system** with the spray device.	Causality to fibrin sealant only a hypothesis. Tisseel fibrin sealant has a special warning and precaution in Summary of Product Characteristics for use with gas and in confined spaces.
Handa, 1989 [64]	Case report	In an observational study fibrin sealant (Beriplast B) was used in 48 places, at 36 neurosurgical operations in 34 patients. In one case where fibrin sealant was applied over the dural surface, **obstruction of an epidural drain** occurred resulting in an epidural haematoma (no further details in abstract).	Spray directly on drain potential obstruction.
Kanazawa, 2010 [65]	Case report	A 65 year old woman underwent surgical craniotomy where arachnoid plasty with fibrin sealant (Beriplast) was completed. Nine days post-surgery the patient underwent abrupt neurological deterioration. Neuroimaging and clinical findings indicated allergic reaction that was successfully treated with steroids. It was hypothesized that components in the fibrin sealant led to the observed **allergic reaction**.	Causality to fibrin sealant only a hypothesis.
Schlenker, 1987 [66]	Controlled trial. One case report	This controlled trial evaluated fibrin sealant (Tissucol) for the prevention of post lumbar puncture headache. Following lumbar puncture, patients were treated with fibrin sealant injected through lumbar needle immediately after dural tap. The first 6 patients were treated uneventfully. The 7th patient, a 58 year old female, developed **aseptic meningitis** treated with antibiotics. Allergy test for fibrin sealant components were negative. No evidence of contamination of trial product. The author suspected chemical irritation caused by component of the fibrin sealant could not be excluded.	Causality to fibrin sealant only a hypothesis.
Wakamoto, 2002 [67]	2 Case reports	*Limited information from abstract in English*: A 56 year old female undergoing microvascular decompression. Eighteen days after procedure the patient was diagnosed with aseptic meningitis. A 30 year old male experienced the same symptoms 15 days after same surgery. Dacron, Goretex and Lyodula were used together with fibrin sealant, the latter to prevent CSF leak. It was suggested that the human fibrinogen was the cause of the **aseptic meningitis** that was successfully treated with steroids.	Causality to fibrin sealant only a hypothesis.

When evaluating safety in these complex interventions and in patients with potential comorbidities, it is important to take into consideration the fact that a number of other products, including fibrin sealant, are used during these procedures. As such, direct determination of causality is difficult. Overall the specific adverse events are few and not frequently reported over a period of more than 25 years.

## Discussion

Cerebrospinal fluid does not forgive and a careless dural closure almost certainly will result in a leak. For the surgeon, cerebrospinal fluid leaks are a frustrating complication of neurosurgical procedures, while for the patient these can result in unanticipated morbidity and mortality. Like most complications, cerebrospinal fluid leaks are best managed by prevention of their occurrence. During recent decades, dural closure after routine and emergency neurosurgical procedures has been performed with the use of suitable autologous, heterologous, or many different synthetic materials, and also with biological, semisynthetic, or synthetic glues. Such materials have been used individually or combined according to different techniques reported in the literature.

The introduction of fibrin sealants has resulted in the rate of postoperative CSF leakage being dramatically reduced thus reducing also the costs related to the management of CSF leak complications and the length of postoperative hospitalization. Fibrin sealants are widely used for a variety of neurosurgical indications such as dural closure and/or reinforcement

With this systematic literature review we aimed to evaluate the evidence available in the literature and relate it to our experience with fibrin sealants in neurosurgery. Our goal was to evaluate whether the clinical clinical data for fibrin sealants support their use in dura sealing, either to prevent acute CSF leaks (perioperatively) in patients after neurosurgery or to prevent/treat persistent CSF leaks (postoperatively or with instrumentation) with regards to efficacy and safety of the products. This extensive literature search identified only a few randomized clinical trials that provide a high level of evidence regarding the efficacy and safety of fibrin sealants used for these indications. The literature search identified several lower quality studies including case series and reports that were included in our analysis, with these representing the majority of studies evaluated and a limitation to the conclusions that may be drawn from the findings.

In this systematic review the best quality study was a large randomized controlled trial that evaluated the perioperative application of fibrin sealants directly to the suture line to stop acute CSF leaks in patients undergoing neurosurgical procedures. The definition of a perioperative acute CSF leak is straightforward and simple and evaluated on the spot by the neurosurgeon. In the largest randomized controlled trial identified in this review, liquid fibrin sealant was applied directly to the suture line with a clear superior efficacy as it provided watertight closure of the durotomy in 92% versus 28% in the suture only group [29]. This feature allows the final closure of patient and perhaps a reduced surgical time, however this is not supported by the available data. However, these findings were not consistent with a reduced frequency of postoperative clinically relevant CSF leaks.

The definition of postoperative CSF leaks varied widely from extensive use of radio imaging with the identification of even small non clinically relevant fluid accumulations [29] to only evaluating clinical relevant CSF leaks (most of the reported studies). The clinical relevance of evaluating the effect of fibrin sealants on fluid accumulations detected with imaging such as magnetic resonance is doubtful [29]. The available evidence on the effect of fibrin sealants on clinically relevant postoperative CSF leaks is based mostly on lower evidence case series with the potential of bias. Nevertheless, and with the limitations of the identified clinical studies, fibrin sealants seemed to control the formation of postoperative CSF leaks in the supratentorial/posterior fossa [10, 11, 33, 35, 41, 46, 47, 48, 49, 50, 52], spinal [32, 39, 40, 45, 51, 56] and transsphenoidal [7, 34, 37, 42–44, 55] procedures. Importantly, fibrin sealants were used in a large variety of ways including directly on the suture line, on patches (sutured or non-sutured), together with haemostatic agents such as Surgicel and Gelfoam, and with several autologous tissues. Thus, a potential effect directly from the fibrin sealant used is difficult to confirm.

Only two papers, both case series, discussed the use of fibrin sealants as a treatment of persistent CSF leaks. In patients that had undergone endoscopically anterior skull base repair, high success rates were achieved after the closure of the CSF fistula with a combination of graft plus fibrin sealant plus Surgical [58], although it is difficult to assess the direct effect of fibrin sealant in these cases. In the analysis of 10 patients with persistent CSF fistulas after different operative approaches, fibrin sealant was injected directly into the fistula with full closure of all fistula [42].

The safety profile of fibrin sealant products has been tested over many decades. The randomized controlled trials reported in this systematic literature review [24, 29, 32] were not able to detect specific adverse events with a higher frequency in the fibrin sealant group. In the group of case series analyzed, of which most were without any control groups, no consistent or repeated safety issue was detected. As summarized in Table 7, specific adverse events were reported as early as 1987 in 7 papers identifying 8 events in neurosurgical procedures. Most of these events were only assumed to be related to fibrin sealants. One event of gas emboli occurred after the use of the product with disregarding of the warnings for such risk [63]. One case of anaphylactic shock occurred after reexposure to the product and was confirmed with antibodies to product component [61].

The use of fibrin sealants imparts the possibility of local and/or systemic toxicity, allergic reactions, transmissible diseases, zoonosis, etc. Nevertheless, no such transmissible diseases have been reported and, in general, adverse effects have been very rarely reported in the pertinent literature, of which most were speculative.

Limitations to his systematic literature review include the limited number of randomized controlled studies that have been conducted to evaluate the effect of fibrin sealants on intraoperative dura closure as well as on postoperative cerebrospinal fluid leakage. It is unfortunate that the majority of what we identified was observational data including case series information that is inherently flawed. The need for high quality studies and clinical evidence in neurosurgery is essential. Recently, Mansouri and colleagues (2016) analyzed the quality of neurosurgical randomized controlled trials and also found a low prevalence of such analyses and a low quality of design and reporting with many study designs not compatible with the stated objectives [68]. While the authors did not imply that randomized controlled trials are not appropriate to neurosurgery evaluations, they did imply that all neurosurgical questions can be answered through a randomized controlled trial and that there is a need for other approaches. In situations where the conduct of a randomized controlled trial is not feasible, a proposed alternative is the conduct of well-designed prospective observational trials that adhere to as many of the principles of sound clinical research as possible [68].

Other limiting factors should be taken into consideration when evaluating the research question. One limitation is how surgery is often practiced. Specifically, surgery due to its nature is more “experience based” and less “evidence based”. While drugs can be administered based on an established dose response relationship, many neurosurgical approaches have to be tailor made during surgery to fit the specific patient needs at the moment. As such, the best approaches in surgery are often published in case series based on a single or few surgeons experiences. These important techniques may be valuable for other surgeons to follow in order to treat the patients optimally although this is based on experience rather than evidence. In this systematic literature review we have to respect this paradigm and, as such, included data from publications other than randomized controlled trials such as from case series. Additionally, several types of fibrin sealants have been allowed to be evaluated in this review ranging from liquid fibrin sealant to that of the patch formulation, with this therapeutic heterogeneity impacting the generalizability of the findings. As the active components are the same (thrombin plus fibrinogen) the added patch might add a theoretical advantage over liquid fibrin sealant when large dura gaps are being treated. Fibrin sealant is rarely used alone and oftentimes allogeneic patches are used to reinforce the suture line and to close gaps. The allowance of all types of fibrin sealant type products did not allow for any comparisons between products, which may limit the overall significance of this work for the neurosurgical audience. Further, the degree of clinical heterogeneity in patients (age, sex, race, medical condition) and the use of secondary treatments (ie, medical therapies, interventional strategies) as well as the neurosurgical characteristics and the definitions of CSF leak intraoperatively versus postoperatively also limit our ability to generalize our findings. It is well established that the incidence of CSF leaks are dependent on patients, their disease, and neurosurgical characteristics and that the effects of fibrin sealants may differ significantly based on the type of surgery (ie, spinal, posterior fossa, pituitary). An additional limitation of our analysis was that very different techniques are used based on the experience of the neurosurgeon. These differences might influence the strength of concluding a potential observed effect of fibrin sealants in treating/preventing CSF leaks.

A previous meta-analysis has demonstrated that fibrin sealants either reduced blood loss or reduced the time to achieve haemostasis, and both such indicators were positively associated with a good surgical outcome [14]. This systematic literature review has evaluated the use of fibrin sealants to treat/reduce CSF leaks and the results seemed to indicate a reduction in intraoperative and postoperative fluid collections with an acceptable safety profile with the potential of reduced infection, wound problems, pseudomeningocele and pneumocephalus. Furthermore, this may prove to be cost-effective by reducing the incidence of CSF leakage. The strength of evidence identified in this systematic literature review can be strengthened further through additional well-designed and controlled clinical trials.

In summary, we found that of the 32 clinical studies identified, three were randomized controlled trials evaluating the efficacy of fibrin sealants in dura sealing (n = 149 exposed to fibrin sealant). The effect of fibrin sealants in providing watertight closure of the dura suture line was demonstrated in one high quality randomized controlled trial. While there was a significantly higher rate of dura closure in the fibrin sealant than control group (92.1% versus 38.0%), it is notable that the fibrin sealant group had a higher rate (although not statistically significant) of postoperative cerebrospinal fluid leakage (6.7% versus 2.0%, respectively). Most clinical trials evaluated the effect of fibrin sealants in prevention of CSF leaks due to its sealing effect in very different surgical approaches. These are mostly lower level evidence studies that, with caution, may suggest a possible effect of fibrin sealants in preventing postoperative CSF leaks. Two small clinical studies (case series) evaluated the effect of using fibrin sealants in treating persisting leaks without firm conclusions on efficacy. Safety data was presented in the identified citations (including two citations reporting on a single trial) identified clinical studies where a total of 2935 patients had been exposed to fibrin sealants during a variety of neurosurgical procedures. Overall the safety profile with fibrin sealant usage is acceptable. Specific adverse reports where fibrin sealants have been used for dura sealing are very few with just 8 cases, most being of a speculative nature, reported since 1987.

In this review, the majority of identified literature related to the use of the fibrin sealant, Tisseel. This is not unexpected as it has been marketed for over 3 decades. Overall, among the reviewed literature, it was evaluated in one report of acute CSF leaks [36], one randomized controlled trial [29], 11 case series of CSF leak prevention [7, 10, 11, 37, 40, 42, 45, 46, 48, 49, 50, 57], and in one subgroup of patients treated for persistent CSF leaks [42]. The extrapolation of the data derived from Tisseel/Tissucol studies to other fibrin sealants should be done with some degree of caution. Future well-designed and well-powered randomized controlled trials involving Tisseel as well as the other fibrin sealants will strengthen our ability to draw firm conclusions regarding the effectiveness of these agents. In the meantime, the availability and data derived from over 30 years of Tisseel use suggests that it may be considered as a standard part of the “surgical toolbox” when performing neurosurgical procedures where dura sealing is required.

## Conclusions

The overall conclusion of our systematic literature review are that fibrin sealants used in neurosurgery may be beneficial in providing a watertight closure of the dura suture line and may have an effect in preventing CSF leaks with an acceptable safety profile. We caution that this is derived from findings of a single randomized controlled trial that was carried out under specific settings in patients undergoing elective craniotomy or craniectomy for pathological processes in the posterior fossa or in the supratentorial region and had demonstrated persistent CSF leakage from a primary attempt at suture closure of the dural incision. We emphasize that data from well-designed and powered randomized clinical trials are needed to further support these findings and firmly establish the efficacy of fibrin sealants providing intraoperative waterproof dura sealing as well as in limiting postoperative cerebrospinal leakage.

## Supporting Information

S1 PRISMA 2009 Checklist Prisma 2009 checklist completed form. *Modified from*: Moher D, Liberati A, Tetzlaff J, Altman DG, The PRISMA Group (2009). Preferred Reporting Items for Systematic Reviews and Meta-Analyses: The PRISMA Statement. PLoS Med 6(6): e1000097. doi:10.1371/journal.pmed1000097.
(DOC)
