# Correction: Fibrin Sealants in Dura Sealing: A Systematic Literature Review

**DOI:** 10.1371/journal.pone.0175619

**Published:** 2017-04-06

**Authors:** Felice Esposito, Filippo Flavio Angileri, Peter Kruse, Luigi Maria Cavallo, Domenico Solari, Vincenzo Esposito, Francesco Tomasell, Paolo Cappabianca

The second affiliation for the sixth author is not indicated. Vincenzo Esposito is also affiliated with: I.R.C.C.S. Neuromed, Pozzilli, Isernia, Italy.
